# Nanomaterial-enhanced biosensors for traumatic brain injury biomarker detection: a review of analytical performance, machine learning integration, clinical validation, and point-of-care translation

**DOI:** 10.3389/fneur.2026.1876862

**Published:** 2026-08-17

**Authors:** Jacob Wekalao, Tobias Topisia

**Affiliations:** School of Engineering and Technology, National Forensic Sciences University, Gandhinagar, Gujarat, India

**Keywords:** GFAP detection, multiplexed electrochemical sensing, nanomaterial biosensors, point-of-care diagnostics, traumatic brain injury biomarkers

## Abstract

Traumatic brain injury (TBI) affects an estimated 69 million people each year and remains difficult to diagnose rapidly because conventional assessment relies heavily on neurological examination and computed tomography, which may miss subtle injuries or be unavailable in many clinical settings. Blood-based biomarkers, including S100B, glial fibrillary acidic protein (GFAP), ubiquitin carboxy-terminal hydrolase L1 (UCH-L1), and neurofilament light chain (NfL), provide measurable indicators of glial, neuronal, and axonal injury across different stages of TBI. Nanomaterial-based biosensors have emerged as promising platforms for detecting these biomarkers with high sensitivity, rapid response, and potential for point-of-care testing. This review evaluates recent advances in electrochemical, optical, and emerging biosensor technologies for TBI biomarker detection, with emphasis on analytical performance, clinical validation, and translational potential. The review examines the contributions of gold nanoparticles, graphene derivatives, carbon nanotubes, MXenes, and related nanomaterials to improving signal transduction, surface functionalization, and detection sensitivity in immunosensors and aptasensors. Key analytical parameters, including limit of detection, linear dynamic range, assay time, and selectivity, are assessed against clinically relevant thresholds such as the FDA-cleared GFAP cutoff of 22 pg./mL and the UCH-L1 cutoff of 327 pg./mL. Progress in multiplex biosensing, artificial intelligence-assisted data analysis, and portable diagnostic platforms is also discussed. Although many nanomaterial-enabled biosensors achieve analytical performance well below clinical detection thresholds, most have been validated only in buffer solutions or spiked biological samples, with limited evaluation in patient cohorts. Future progress will depend on robust clinical validation, standardized performance assessment, scalable manufacturing, and regulatory alignment to support the translation of TBI biosensors into routine point-of-care practice.

## 1 Introduction

Traumatic brain injury (TBI) is a major global health challenge, affecting tens of millions of people annually and contributing substantially to mortality, disability, and healthcare costs (1–5). Early diagnosis is essential to prevent secondary brain injury, yet many patients—particularly those with mild TBI (mTBI), which accounts for approximately 80–90% of all cases—are difficult to diagnose because symptoms are nonspecific and conventional imaging often appears normal (6–10). In low- and middle-income countries, limited access to computed tomography (CT) further delays diagnosis and treatment, emphasizing the need for rapid, affordable, and portable diagnostic technologies (11–15).

CT remains the primary imaging modality for acute TBI because of its speed and ability to detect intracranial hemorrhage and mass lesions requiring emergency intervention (16–20). However, CT has limited sensitivity for diffuse axonal injury and other non-hemorrhagic abnormalities commonly associated with mTBI, while repeated radiation exposure poses additional concerns. Magnetic resonance imaging (MRI) provides greater sensitivity for detecting microstructural brain injury but is expensive, time-consuming, and often unavailable in emergency or resource-limited settings. Moreover, both CT and MRI provide only structural snapshots and cannot monitor the dynamic biochemical processes underlying secondary brain injury (21–25).

Blood-based biomarkers have emerged as valuable complementary tools for TBI diagnosis and monitoring. Biomarkers such as S100B, glial fibrillary acidic protein (GFAP), ubiquitin carboxy-terminal hydrolase-L1 (UCH-L1), neurofilament light chain (NfL), tau proteins, neuron-specific enolase, spectrin breakdown products, and inflammatory cytokines reflect different aspects of neuronal and glial injury (26–30). The clinical significance of biomarker testing was established by the FDA approval of the Banyan Brain Trauma Indicator in 2018 and the i-STAT TBI panel in 2021, demonstrating the feasibility of blood-based diagnostics for TBI.

Nanomaterial-based biosensors have emerged as promising platforms for rapid, sensitive, and point-of-care detection of these biomarkers. Nanomaterials including gold nanoparticles, graphene derivatives, carbon nanotubes, MXenes, and related nanostructures enhance biosensor performance through increased surface area, improved electron transfer, and superior optical and electrochemical properties, enabling detection of biomarkers at clinically relevant concentrations (31–35). Recent advances have also introduced multiplexed biosensors capable of simultaneously measuring multiple biomarkers, while machine learning algorithms improve signal processing, biomarker interpretation, and diagnostic accuracy.

Despite remarkable analytical progress, significant translational challenges remain. Many reported biosensors have been validated only in buffer solutions or biomarker-spiked samples, with relatively few evaluated using clinical patient specimens or compared against established reference assays. Furthermore, existing reviews rarely relate analytical performance directly to clinically relevant biomarker thresholds or comprehensively assess validation maturity and multiplexed sensing strategies. Therefore, this review focuses on three key aspects: correlating biosensor performance with clinical biomarker concentrations, evaluating validation across biological matrices and patient samples, and examining advances in multiplexed nanomaterial-based biosensors integrated with artificial intelligence for next-generation TBI diagnosis (36–40).

## 2 TBI epidemiology, biomarkers, and pathophysiology

### 2.1 Global epidemiology of traumatic brain injury

**Figure 1:**
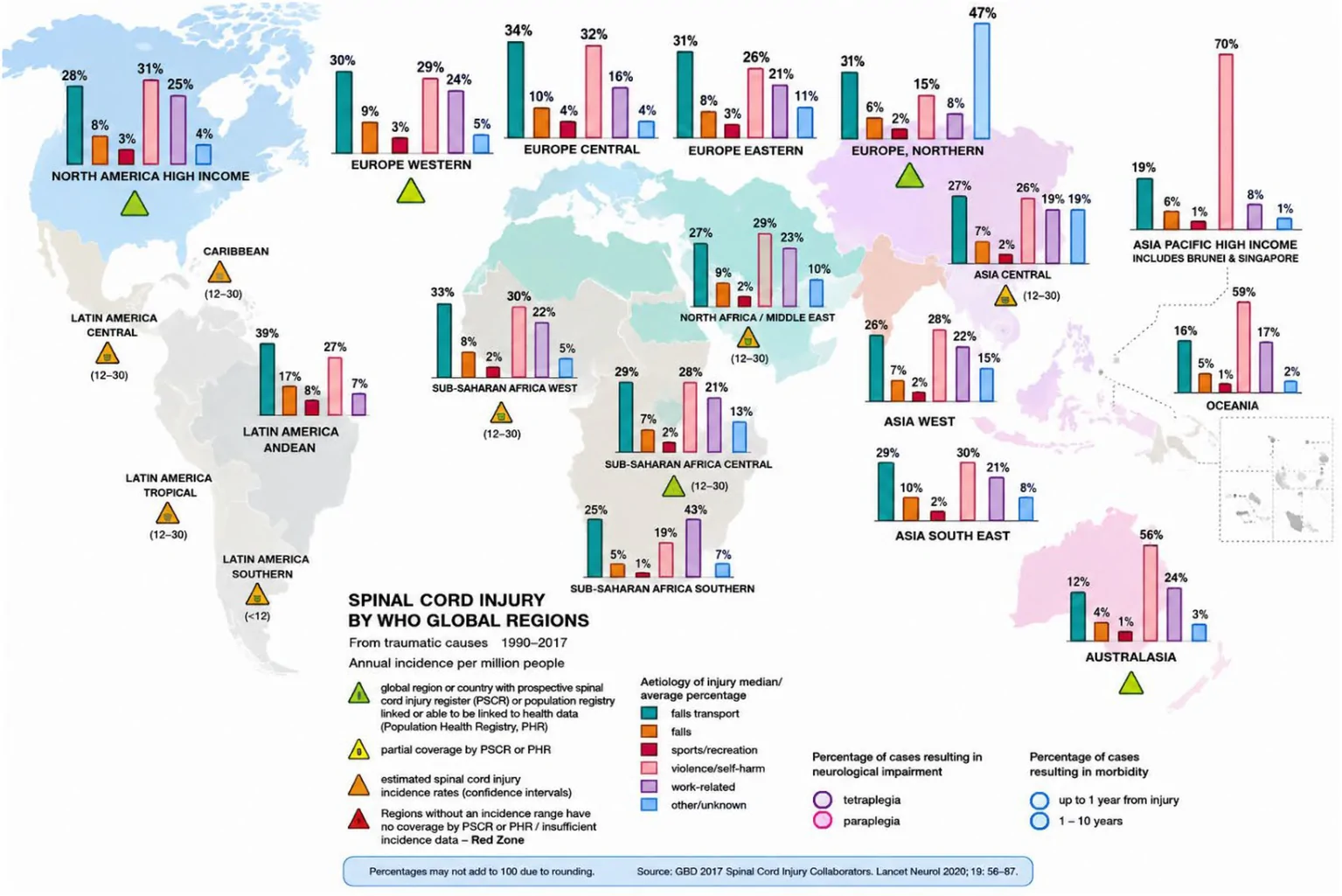
Spinal cord injury by WHO Global Regions from traumatic causes (49).

Traumatic brain injury (TBI) is a leading cause of death and disability worldwide, affecting an estimated 69 million people annually and accounting for approximately 2.87 million emergency department visits, hospitalizations, and deaths each year in the United States, with an economic burden exceeding US$76 billion (41–44). The true incidence is likely higher because many cases of mild TBI remain undiagnosed due to underreporting, nonspecific symptoms, and the lack of objective diagnostic criteria. TBI epidemiology varies across age groups, with road traffic accidents and interpersonal violence predominating among young adults, falls representing the major cause in older adults, and sports- and military-related injuries contributing significantly to research on repetitive mild TBI and chronic traumatic encephalopathy (45, 46). Low- and middle-income countries bear a disproportionate share of TBI-related mortality because of increasing road traffic injuries, limited access to computed tomography, shortages of neurosurgical services, and underdeveloped trauma systems, resulting in delayed diagnosis and poorer outcomes (47). Beyond the acute injury, TBI frequently leads to persistent cognitive impairment, psychiatric disorders, epilepsy, reduced functional independence, and chronic neurodegeneration, creating substantial long-term healthcare and socioeconomic burdens. These challenges highlight the importance of developing rapid biomarker-based diagnostic technologies that enable early diagnosis, timely intervention, and improved patient management across diverse healthcare settings (48). Figure 1 depicts the Spinal cord injury by WHO Global Regions from traumatic causes (49).

### 2.2 Major biomarkers for traumatic brain injury

**Figure 2:**
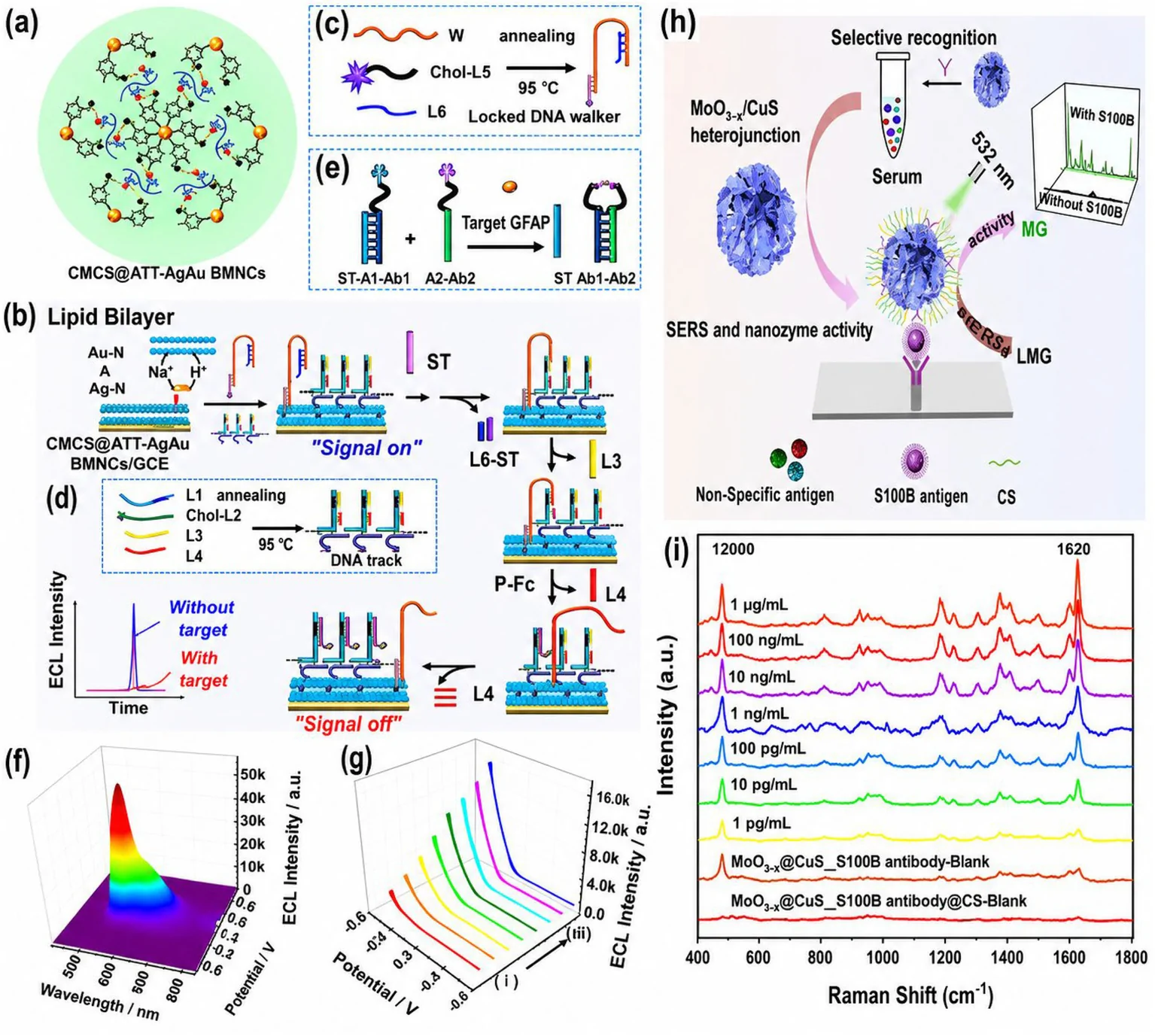
Schematic illustration of a dual ECL-SERS biosensor based on CMCS@ATT-AgAu bimetallic nanoclusters and MoO₃_−x_/CuS heterojunctions integrating DNA walker signal switching and specific antigen recognition for GFAP and S100B detection alongside corresponding ECL and Raman characterization spectra (189). **(a)** schematic of CMC@ATT-AgAu BMNCs nanoparticles; **(b–e)** stepwise assembly and function diagrams for a lipid bilayer-based biosensor with DNA walkers; **(f–g)** 3D graphs of ECL intensity versus wavelength and potential; **(h)** diagram of S100B antigen detection using MoO₃/CuS heterojunction nanozyme with serum and Raman response; and **(i)** overlaid Raman spectra representing intensity at different S100B antigen concentrations.

S100B, GFAP, UCH-L1, and neurofilament light chain (NfL) are the most clinically validated biomarkers for traumatic brain injury (TBI), reflecting complementary aspects of astrocytic, neuronal, and axonal damage, with distinct molecular origins and temporal release profiles that support different diagnostic applications (50, 51). S100B is an astrocytic calcium-binding protein that peaks within 2–6 h after injury and is widely used for early exclusion of CT-positive lesions in mild TBI, although its specificity is limited by extracranial expression (52, 53). GFAP, an astrocyte-specific intermediate filament protein, provides greater central nervous system specificity, remains elevated for up to 5 days, and forms part of the FDA-cleared Banyan Brain Trauma Indicator (BTI) using a diagnostic threshold of 22 pg./mL to predict CT-detectable intracranial injury (54). UCH-L1, a neuron-specific deubiquitinating enzyme, appears in blood within approximately 1 h after injury and, together with GFAP, constitutes the FDA-approved Banyan BTI panel, using a clinical threshold of 327 pg./mL for early detection of intracranial lesions (55). In contrast, NfL is a structural axonal protein that remains elevated for weeks to months after injury, making it particularly valuable for monitoring subacute and chronic neuroaxonal damage, repetitive mild TBI, and long-term neurological outcomes, although age-adjusted reference values are required for accurate interpretation (56). Table 1 summarizes the comparative characteristics of the major TBI biomarkers, while Figure 2 illustrates a representative dual electrochemiluminescence (ECL)-surface-enhanced Raman scattering (SERS) biosensor based on CMCS@ATT-AgAu bimetallic nanoclusters and MoO₃_−x_/CuS heterojunctions.

**Table 1:** Comparative characteristics of major traumatic brain injury biomarkers, including molecular properties, biological origin, temporal kinetics, regulatory thresholds, and primary clinical applications (192–196).

Biomarker	Molecular weight	Primary cell source	Peak in serum	Half-life	FDA threshold	Primary diagnostic role
S100B	21 kDa	Astrocytes, Schwann cells	2 to 6 h	Approximately 97 min	0.10 μg/L*	Acute exclusion of CT-positive lesions
GFAP	50 kDa	Astrocytes (central nervous system)	1 to 2 h, remains elevated for 72 to 120 h	Approximately 24 h	22 pg./mL	Acute diagnosis and outcome prediction
UCH-L1	25 kDa	Neurons	Less than 1 h	Approximately 7 h	327 pg./mL	Early acute diagnosis
NfL	68 kDa	Axons	Several days	1 to 3 weeks	No FDA-approved cutoff	Monitoring subacute, chronic, and repetitive TBI

*The 0.10 μg/L threshold is recommended by the Scandinavian Neurotrauma Committee. Other clinical guidelines use different decision thresholds.

Following brain injury, brain-enriched proteins are released into accessible body fluids — including blood (serum and plasma), cerebrospinal fluid (CSF), and, in emerging non-invasive approaches, saliva, tears, and sweat—where they can be quantified as molecular indicators of glial, neuronal, and axonal injury. Because CSF is a central-nervous-system-derived compartment rather than a peripheral one. Clinical use of these biomarkers requires detection methods capable of multiplexed measurement at very low concentrations across different body fluids, including blood-based matrices (serum and plasma) and emerging non-invasive samples such as saliva, tears, and sweat.

GFAP and NfL concentrations are strongly assay-dependent, and reported values are not directly comparable across analytical platforms due to major differences in sensitivity, calibration, and detection limits (57–59). For GFAP, the FDA-cleared 22 pg./mL threshold from the Banyan Brain Trauma Indicator is based on chemiluminescent immunoassays (later adapted to Abbott ARCHITECT and i-STAT platforms), whereas sub-picogram baseline levels are measured using single-molecule array (Simoa) digital immunoassays, with conventional ELISA generally lacking sufficient sensitivity for low-level detection in healthy individuals or mild TBI (60, 61). Similarly, clinically used NfL reference ranges are derived almost exclusively from Simoa-based assays because standard ELISA cannot reliably detect its low physiological concentrations, and all reported values require age-adjusted, platform-specific interpretation (62, 63). Unless otherwise stated, GFAP values refer to blood (serum/plasma) measurements used in FDA-cleared systems, not CSF, as CSF GFAP is substantially higher and kinetically distinct, and thresholds are not interchangeable across compartments (64).

### 2.3 Pathophysiological cascades

Traumatic brain injury (TBI) develops through two overlapping phases: primary injury and secondary injury. Primary injury occurs at the moment of impact and results from direct mechanical forces that cause tissue deformation, contusions, diffuse axonal injury, and disruption of the blood–brain barrier. These events produce immediate damage to neurons, glial cells, and cerebral blood vessels, allowing brain-derived proteins to enter the bloodstream. Because primary injury reflects irreversible mechanical damage, treatment focuses on limiting the biological processes that follow rather than reversing the initial tissue destruction (65–67).

**Figure 3:**
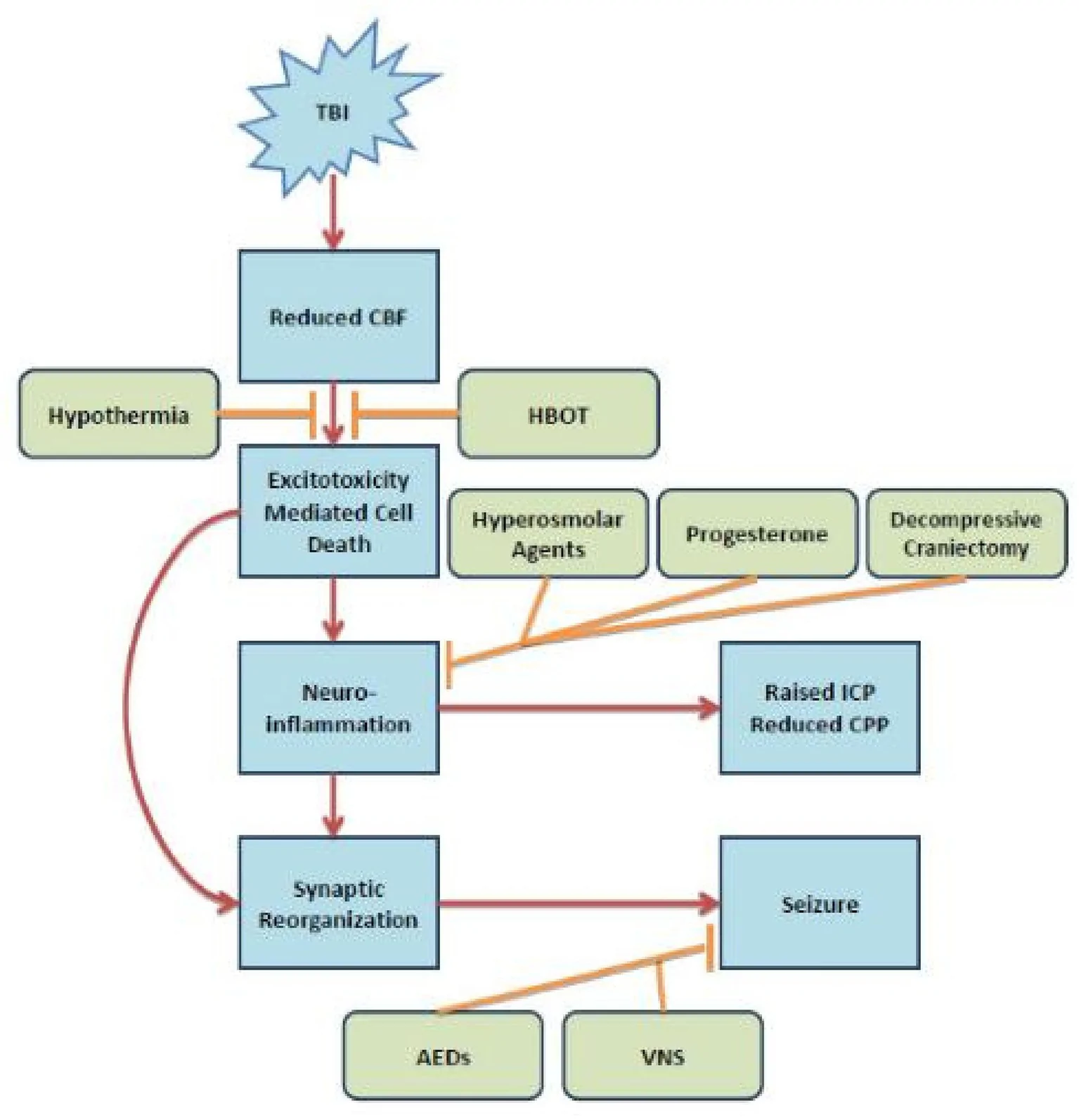
The schematic pathway illustrating secondary injury cascades following traumatic brain injury (TBI).

Secondary injury evolves over hours to days through a series of interconnected processes that include excitotoxicity, metabolic dysfunction, neuroinflammation, blood–brain barrier disruption, cerebral edema, and programmed cell death. Excessive glutamate release and calcium influx impair cellular metabolism, while activated microglia release inflammatory cytokines that contribute to ongoing tissue injury. Increased blood–brain barrier permeability promotes edema and reduces cerebral perfusion, further worsening ischemic damage. Neuronal loss occurs through both necrosis and apoptosis, extending tissue damage beyond the initial trauma. These secondary mechanisms provide the biological basis for circulating biomarkers such as S100B, GFAP, UCH-L1, and neurofilament light chain (NfL), which support diagnosis, prognosis, and monitoring of TBI progression (68, 69). Figure 3 demonstrates the Schematic pathway illustrating secondary injury cascades following traumatic brain injury (TBI), including reduced cerebral blood flow (CBF), excitotoxic cell death, neuroinflammation, and synaptic reorganization, alongside targeted therapeutic interventions that modulate each pathological stage to mitigate elevated intracranial pressure (ICP), reduced cerebral perfusion pressure (CPP), and post-traumatic seizures.

### 2.4 Current diagnostic modalities

**Figure 4:**
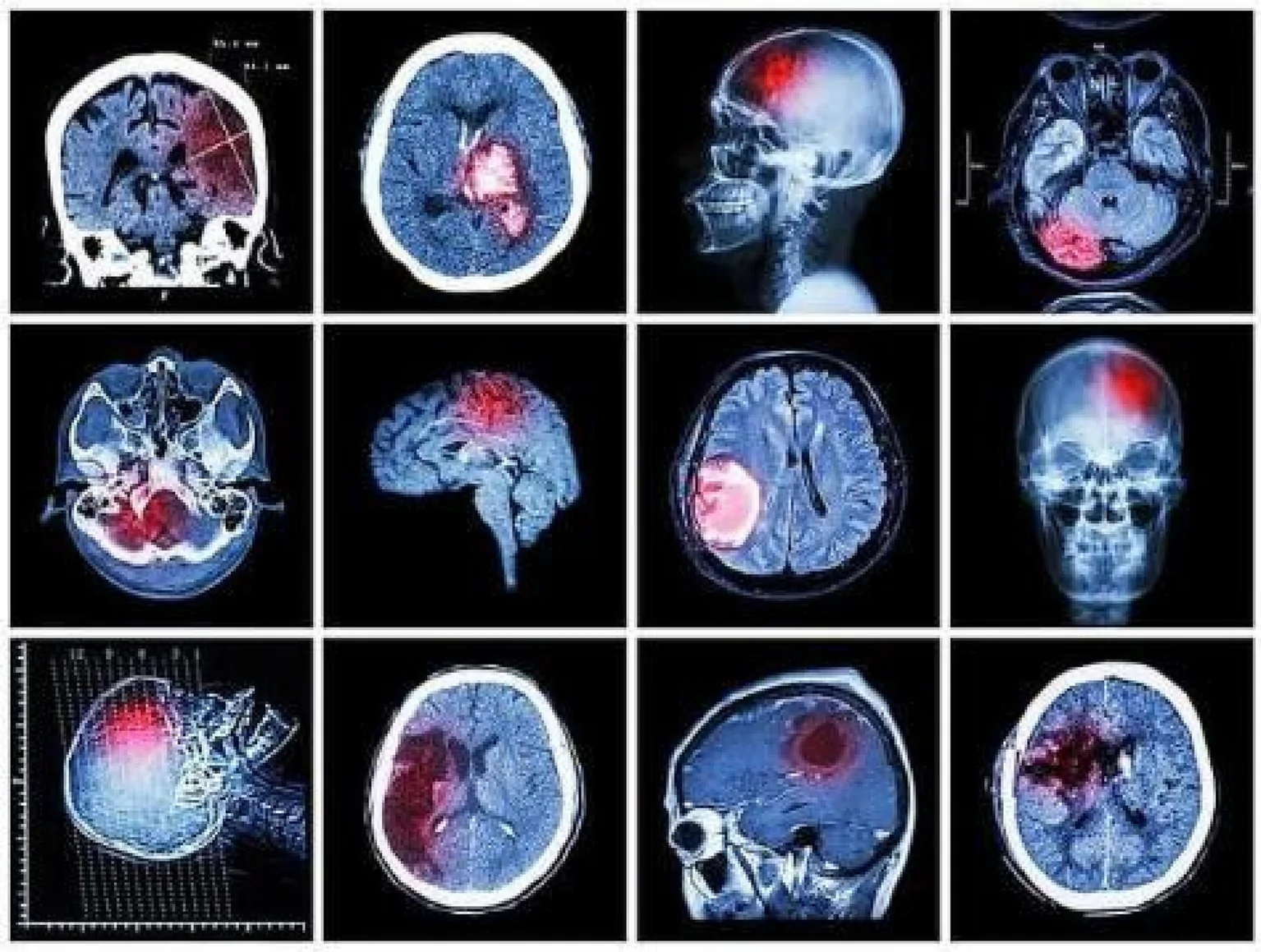
Brain injury tests, procedures, and outlook for TBI patients (190).

Current TBI clinical evaluation integrates neurological examination, injury mechanism assessment, and neuroimaging, with the Glasgow Coma Scale (GCS) used for severity grading but limited in sedated/intubated patients and unable to resolve specific injury types. CT decision rules (Canadian CT Head Rule, New Orleans Criteria, and NEXUS II) improve imaging triage in mild TBI, while sports tools (SAC, SCAT, and King-Devick) assess cognition but are subjective and non-pathological; in severe cases, intracranial pressure monitoring and cerebral microdialysis provide invasive physiological/metabolic data but are restricted to specialized centers, underscoring the need for rapid biosensors for molecular TBI assessment (70–72). Emerging biosensors target both established biomarkers (S100B, GFAP, UCH-L1, and NfL) and additional candidates including tau, NSE, spectrin breakdown products, and inflammatory cytokines (IL-6, IL-8, IL-10, TNF-*α*, and HMGB1), with multiplex detection offering improved diagnosis, prognosis, and monitoring potential (73–75) (Figure 4).

Table 2 maps the limit of detection, linear range, assay time, and sample matrix of each biosensor platform against the established clinical decision thresholds for S100B, GFAP, and UCH-L1. It.

**Table 2:** Clinical biomarker concentration ranges by TBI severity and time post-injury (197–202).

Biomarker	Healthy/uninjured	Mild TBI (mTBI)	Moderate TBI	Severe TBI	Peak timing	Half-life/detection window	Clinical decision threshold	Source
S100B	<0.10 μg/L	193 ± 77 pg./mL (0–24 h)	543 ± 159 pg./mL	1,883 ± 825 pg./mL	2–6 h post-injury	~97 min; useful 0–12 h	0.10 μg/L (SNC, 3–6 h); 0.15 μg/L (other guidelines)	Clinical prospective cohort data
GFAP	<1–2 pg./mL	Elevated within 1–2 h; rises progressively	Markedly elevated	Highly elevated (μg/L range)	1–3 h; sustained	72–120 h elevation	22 pg./mL (FDA-BTI, Banyan)	Banyan BTI regulatory submission
UCH-L1	<30 pg./mL	Elevated by 1 h post-injury	Moderate elevation	High elevation (ng/mL range)	~1 h post-injury	~7 h serum half-life; peak useful 0–12 h	327 pg./mL (FDA-BTI, Banyan)	Banyan BTI regulatory submission
NfL	Age-dependent; typically 2–15 pg./mL	51 ± 12 pg./mL (24–36 h)	100 ± 32 pg./mL	252 ± 78 pg./mL	Days to weeks post-injury	Elevated 1–3 + weeks; persists months	No FDA threshold; SIMOA LOD ~ 1.6 pg./mL (research reference)	Prospective cohort; SIMOA platform
Tau (total)	<10 pg./mL	Elevated; variable	Significantly elevated	Highest elevation; associated with mortality	Hours–days	Days–weeks	No FDA clearance	Research use
p-tau231	Low	Elevated; correlates with CT findings	Elevated	Elevated; associated with MCTC score	Hours–days	Days–weeks	No FDA clearance; research marker	CENTER-TBI data

All concentration values represent approximate ranges from prospective clinical cohort studies and vary with assay platform, timing post-injury, age, and comorbidities, with age-adjusted reference ranges particularly critical for NfL interpretation due to its strong age dependence. Because circulating S100B, GFAP, UCH-L1, and NfL occur at low picogram-per-millilitre levels, conventional ELISA is generally insufficient for reliable quantification (203, 204), and most validated TBI biomarker data instead derive from ultrasensitive laboratory platforms that define current analytical benchmarks. Single-molecule array (Simoa) enables femtogram-per-millilitre detection via digital single-molecule counting and serves as the reference standard for blood GFAP and NfL clinical thresholds (205–207), electrochemiluminescence multi-array (MSD) provides multiplexed intermediate sensitivity using SULFO-TAG–based detection for combined protein panels (208–211), and automated microfluidic ELISA systems (Ella/Simple Plex) offer rapid, low-volume, reproducible near-laboratory testing with improved sensitivity over conventional ELISA (212–215). These platforms underpin key clinical decision thresholds such as 22 pg/mL GFAP and 327 pg/mL UCH-L1, but remain limited by centralized instrumentation, laboratory dependency, and lack of deployability in emergency, sports, and low-resource settings—gaps that nanomaterial-based biosensors aim to address.

## 3 Biosensor fundamentals

A biosensor is an analytical device that combines a biological recognition element with a signal transducer to detect a specific target molecule. The recognition element selectively binds the analyte, while the transducer converts this interaction into a measurable electrical, optical, or mechanical signal (76). For traumatic brain injury (TBI) applications, antibodies remain the most common recognition elements because of their high affinity and specificity for protein biomarkers such as GFAP, S100B, UCH-L1, and neurofilament light chain (NfL). Alternative recognition elements include aptamers, which are synthetic single-stranded DNA or RNA molecules selected through the systematic evolution of ligands by exponential enrichment (SELEX), and molecularly imprinted polymers (MIPs), which contain artificial binding cavities that match the target molecule. Aptamers offer high chemical stability and reproducible manufacturing, while MIPs provide low production cost and long-term stability, making both attractive for point-of-care biosensors (77, 78).

**Figure 5:**
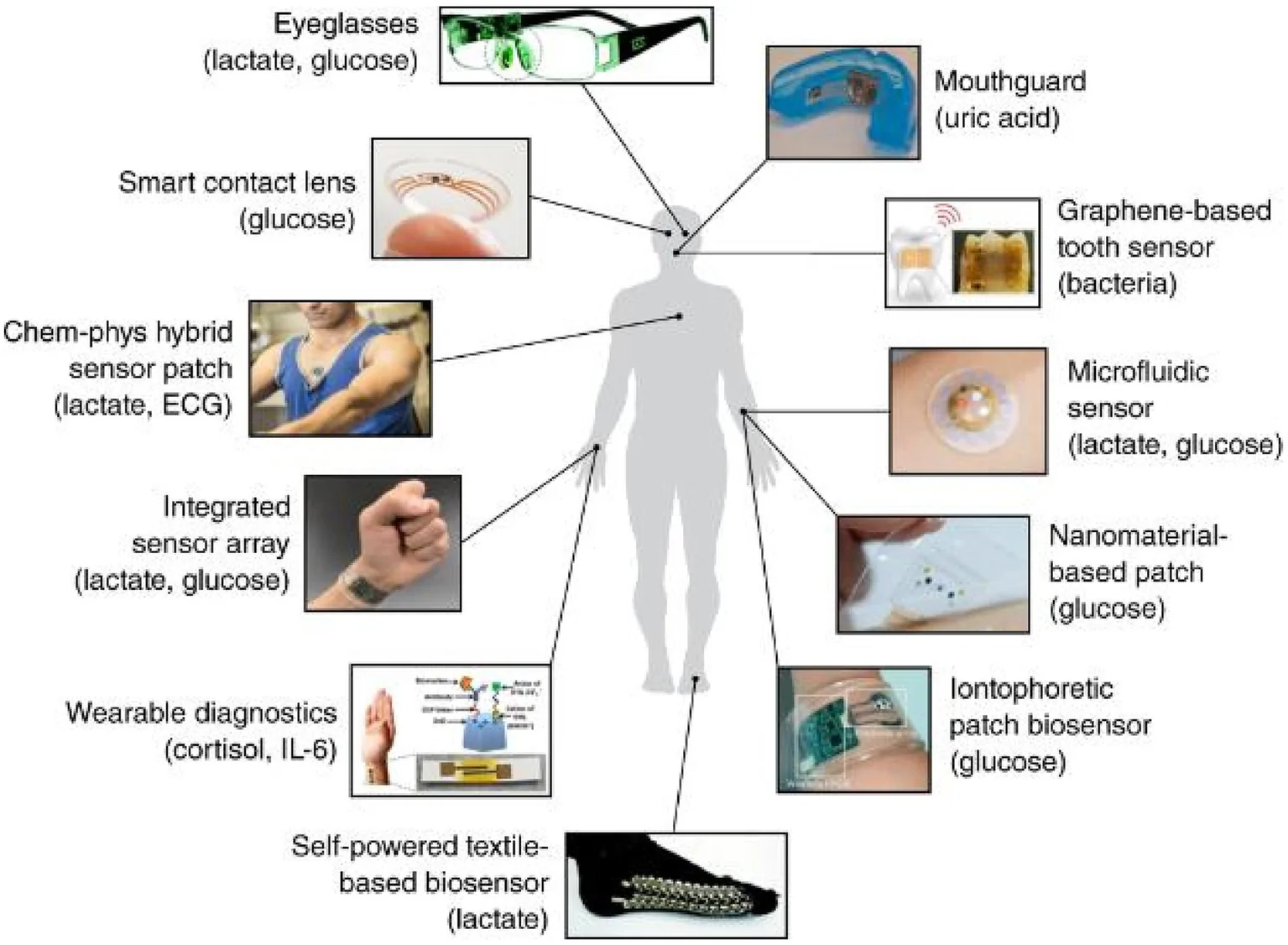
Schematic overview of diverse wearable biosensing platforms distributed across multiple human body sites for non-invasive detection of key physiological biomarkers including glucose, lactate, uric acid, bacteria, cortisol, IL-6, and electrocardiographic signals (191).

The transducer determines how the biomolecular interaction is measured. Electrochemical biosensors detect changes in current, voltage, impedance, or charge at an electrode surface. Optical biosensors monitor changes in light absorption, fluorescence, refractive index, or surface plasmon resonance, whereas acoustic biosensors detect variations in the oscillation frequency of piezoelectric materials following analyte binding (79). Nanomaterials have substantially improved biosensor performance by increasing the available surface area for biomolecule immobilization, enhancing electrical and optical signal transfer, and providing chemically functional surfaces for stable attachment of recognition molecules. These properties improve sensitivity, reduce detection limits, and support miniaturized biosensors suitable for portable and point-of-care diagnostic systems (80). Figure 5 demonstrates the Schematic overview of diverse wearable biosensing platforms distributed across multiple human body sites for non-invasive detection of key physiological biomarkers.

### 3.1 Analytical performance metrics

**Figure 6:**
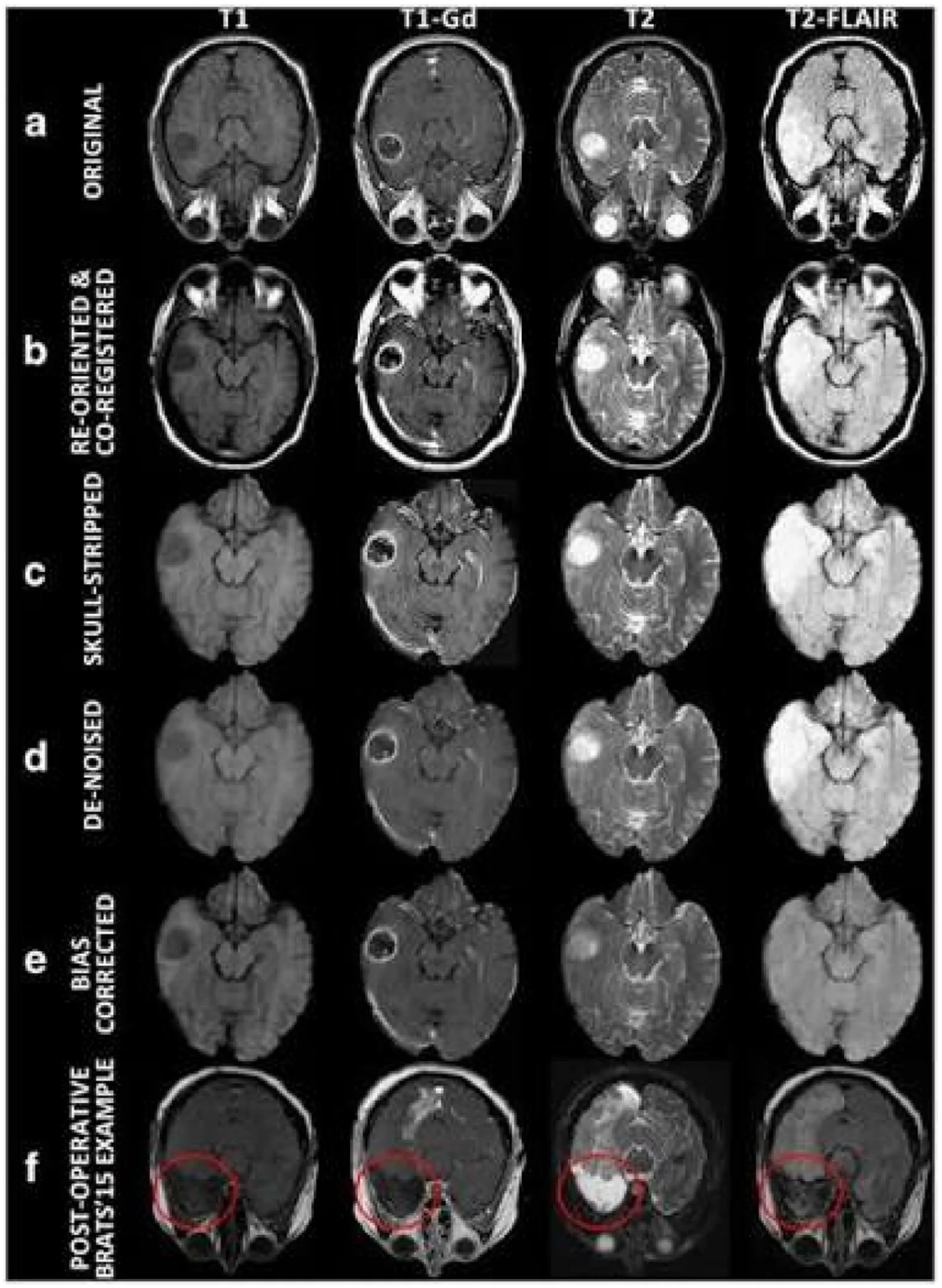
Examples of the original, pre-processed, and post-operative MRI volumes are presented, with intensity non-uniformity correction omitted because it degraded the T2-FLAIR signal (84). **(a)** original MRI volumes; **(b)** re-oriented and co-registered; **(c)** skull-stripped; **(d)** de-noised; **(e)** bias-corrected images across T1, T1-Gd, T2, and T2-FLAIR imaging types; and (f) post-operative examples with red circles highlighting areas of interest.

The analytical performance of a biosensor is evaluated using several quantitative parameters that determine its clinical utility. The limit of detection (LOD) defines the lowest analyte concentration that can be distinguished from background noise, while the limit of quantification (LOQ) represents the lowest concentration that can be measured with acceptable accuracy and precision. The linear dynamic range (LDR) specifies the concentration interval over which sensor output remains proportional to analyte concentration and should encompass the clinically relevant concentration range of the target biomarker. Sensitivity describes the change in sensor signal per unit concentration, whereas selectivity measures the ability to detect the target biomarker in complex biological samples containing abundant interfering proteins. Reproducibility is commonly assessed using intra-assay and inter-assay coefficients of variation, and stability is evaluated through storage and operational testing to determine whether sensor performance is maintained over time (81–83). Figure 6 demonstrates the Examples of the original, pre-processed, and post-operative MRI volumes are presented, with intensity non-uniformity correction omitted because it degraded the T2-FLAIR signal (84).

### 3.2 Transduction modalities

Electrochemical and optical biosensors are the most established platforms for traumatic brain injury (TBI) biomarker detection. Electrochemical sensors convert biomarker binding into changes in current, voltage, or impedance and can operate with enzymatic labels or in label-free formats such as electrochemical impedance spectroscopy. Their low cost, portability, and compatibility with printed electrodes make them well suited for point-of-care devices. Optical biosensors detect changes in fluorescence, color, chemiluminescence, or refractive index, with fluorescence-based assays generally providing the highest sensitivity. Surface plasmon resonance (SPR) enables label-free, real-time measurement of binding kinetics, while nanomaterial-enhanced SPR can reach the low concentrations required for biomarkers such as GFAP and UCH-L1. Förster resonance energy transfer (FRET) biosensors use distance-dependent energy transfer between fluorophores to achieve highly sensitive detection in homogeneous, no-wash assay formats (85–87).

**Figure 7:**
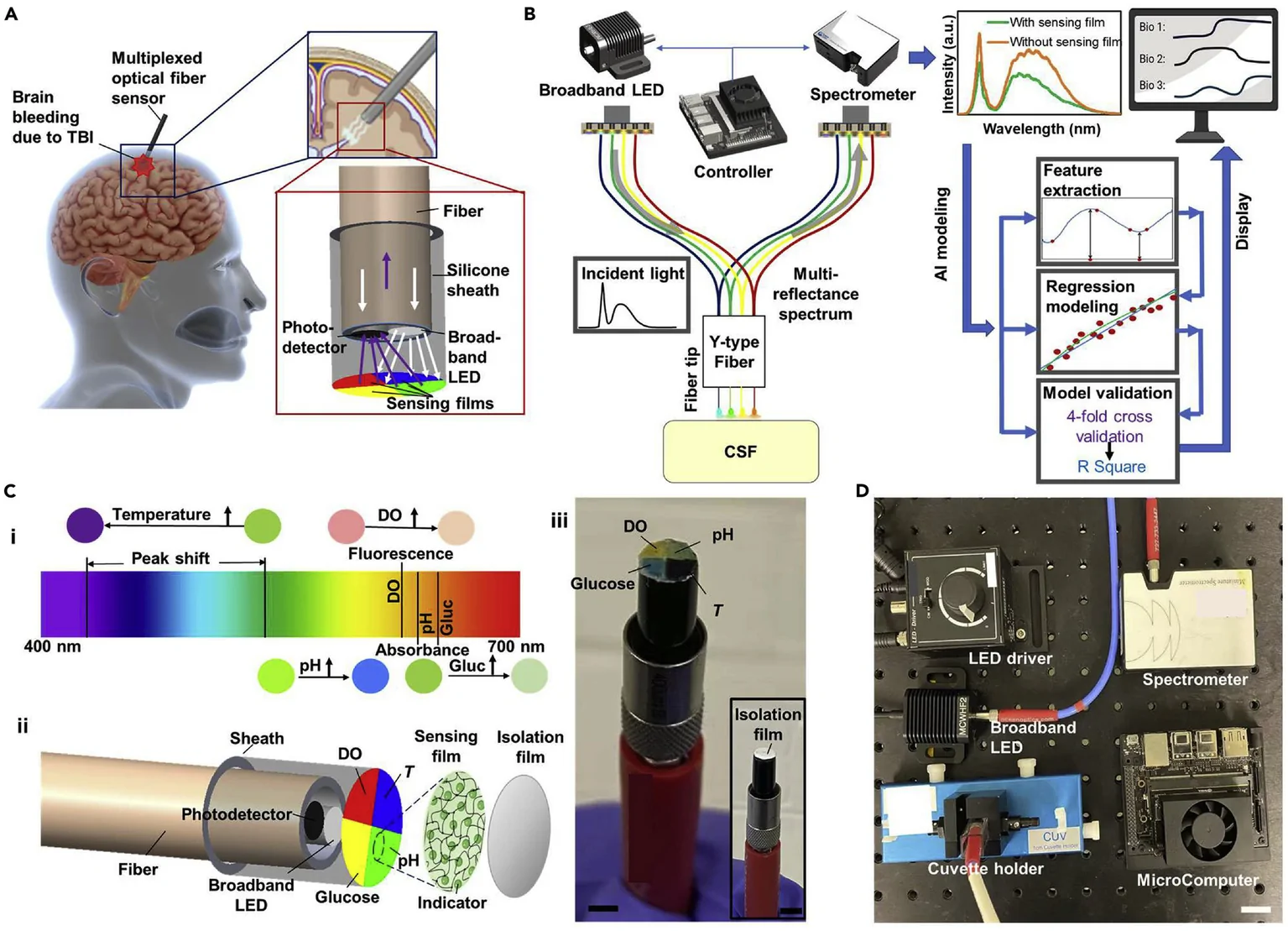
The design, optical setup, sensing principle, and hardware implementation of a multiplexed optical fiber sensor for simultaneous temperature, dissolved oxygen, pH, and glucose detection in cerebrospinal fluid after traumatic brain injury paired with AI spectral modeling for biological signal readout (90). **(A)** anatomical placement and structural details of the fiber sensor; **(B)** experimental setup with broadband LED, spectrometer, controller, fiber tip, and AI modeling workflow; **(C)** wavelength-dependent sensing mechanisms for temperature, dissolved oxygen, glucose, and pH; **(D)** photo of the assembled hardware setup.

Several emerging technologies are extending biosensor capabilities beyond conventional approaches. CRISPR-Cas platforms convert biomarker recognition into amplified nucleic acid signals and can achieve extremely low detection limits with simple readouts. Nanopore sensors detect individual molecules by monitoring ionic current changes through nanoscale pores, enabling digital single-molecule quantification. Terahertz and metasurface biosensors measure changes in dielectric properties and protein-specific spectral signatures, offering both sensitivity and molecular fingerprinting. Graphene field-effect transistor (GFET) biosensors provide label-free, real-time detection by measuring conductivity changes caused by biomolecule binding on graphene surfaces. Together, these emerging modalities aim to deliver faster, more sensitive, and more portable TBI diagnostics suitable for clinical and point-of-care use (88, 89). Figure 7 depicts the design, optical setup, sensing principle, and hardware implementation of a multiplexed optical fiber sensor for simultaneous temperature, dissolved oxygen, pH, and glucose detection in cerebrospinal fluid after traumatic brain injury paired with AI spectral modeling for biological signal readout (90).

Table 3 compares twelve biosensor transduction principles across sensitivity, label requirement, multiplexing capability, point-of-care compatibility, and clinical validation status.

**Table 3:** Transduction modalities: comparative capabilities and limitations for TBI diagnostics (216–220).

Transduction modality	Sensing principle	Key nanomaterials used	Representative LOD range	Label required?	POC compatible?	Multiplex capability	Clinical validation status	Key limitations
Amperometric (voltammetric)	Current change at fixed/swept potential (DPV, SWV, CV)	AuNPs, CNTs, rGO, MXene	fg/mL–pg/mL	Optional (sandwich)	Yes (screen-printed)	Limited (array format)	Mostly buffer/spiked serum	Biofouling; matrix interference
Electrochemical Impedance Spectroscopy (EIS)	Impedance change at electrode–solution interface	rGO, MXene, Au, polymer films	fg/mL–pg/mL	Label-free	Yes	Moderate	Mostly buffer/spiked serum	Calibration drift; non-specific binding
Surface Plasmon Resonance (SPR)	Refractive index change at metal film surface	Gold film	pg/mL–ng/mL	Label-free	Limited (benchtop)	Moderate	Some clinical use (reference assays)	Bulky instrumentation; high cost
Localized SPR (LSPR)	Plasmon shift in nanoparticle extinction	AuNPs, Au nanorods, nanostars	pg/mL	Label-free	Emerging	Limited	Mostly buffer	Limited dynamic range
Fluorescence	Emission intensity or FRET distance-dependence	QDs, Au NCs, graphene QDs	fg/mL–pg/mL	Yes (label)	Emerging (smartphone)	High	Moderate (some clinical)	Photobleaching; autofluorescence
Surface-Enhanced Raman Spectroscopy (SERS)	Raman scattering amplification at hotspots	Ag nanorods, Au/Ag NPs, nSTF	sub-pg/mL	Optional	Emerging	High (spectral encoding)	Limited; mostly pre-clinical	Reproducibility; substrate fabrication cost
Graphene Field-Effect Transistor (GFET)	Conductance change on graphene channel	Monolayer graphene	fg/mL (400 aM GFAP)	Label-free	Emerging	Moderate	Clinical plasma (GFAP demonstrated)	Fabrication complexity; Debye screening
CRISPR-Cas (Cas12a/Cas13a)	Nucleic acid-triggered collateral cleavage amplification	AuNF, nanocomposites (Cas + EChem)	aM–fM range	Signal label	Emerging	High (multiplexable)	Pre-clinical; no TBI-specific clinical data	Requires nucleic acid target or conversion step
Nanopore	Ionic current disruption through nanoscale pore	Solid-state/biological pores	Single-molecule digital	Label-free	Emerging (portable)	Moderate	Pre-clinical	Pore fabrication; data analysis complexity
Terahertz/Metasurface	Dielectric property and resonance frequency shift	Graphene, MXene, gold metasurface	sub-pg/mL (refractive index)	Label-free	Emerging	High (spectral fingerprinting)	Pre-clinical; no clinical TBI data	Bulky THz source; cost
Lateral flow (LFIA)	Colorimetric/fluorescent signal on nitrocellulose	Colloidal gold NPs, fluorescent NPs	pg/mL–ng/mL (higher than above)	Yes (label)	Yes (highest)	Low (usually 1–2 analytes)	Highest clinical maturity (S100B in Europe)	Higher LOD; semi-quantitative only
Colorimetric (smartphone)	Color change detectable by naked eye or camera	Metal NPs, enzyme mimics (nanozymes)	ng/mL rang					

## 4 Biosensing platforms by biomarker

S100B biosensors have advanced from conventional gold and carbon electrodes to nanomaterial-enhanced platforms incorporating gold nanoparticles, graphene derivatives, carbon nanotubes, MXenes, and hybrid nanocomposites. Most devices use sandwich immunoassays with anti-S100B antibodies as the recognition element, although aptamer-based and molecularly imprinted polymer (MIP)-based sensors have also been developed. Electrochemical biosensors detect S100B through changes in current or impedance following antigen–antibody binding, while optical platforms monitor changes in localized surface plasmon resonance (LSPR). Nanomaterials improve sensor performance by increasing the surface area available for biomolecule immobilization, accelerating electron transfer, and enhancing signal generation (91–93).

Recent S100B biosensors have demonstrated detection limits ranging from approximately 10 to 100 fg/mL for electrochemical platforms and 0.5 to 5 pg./mL for optical systems, providing sensitivity well below clinically relevant S100B concentrations. Many electrochemical sensors achieve broad linear detection ranges that extend from picogram to nanogram levels, with assay times of approximately 20 to 60 min. Although these analytical results are promising, most studies have evaluated sensor performance using buffer solutions or biomarker-spiked serum rather than samples collected from patients with traumatic brain injury. Only a limited number of studies have compared biosensor measurements with established clinical immunoassays using appropriate statistical methods, limiting evidence for clinical equivalence (94).

**Figure 8:**
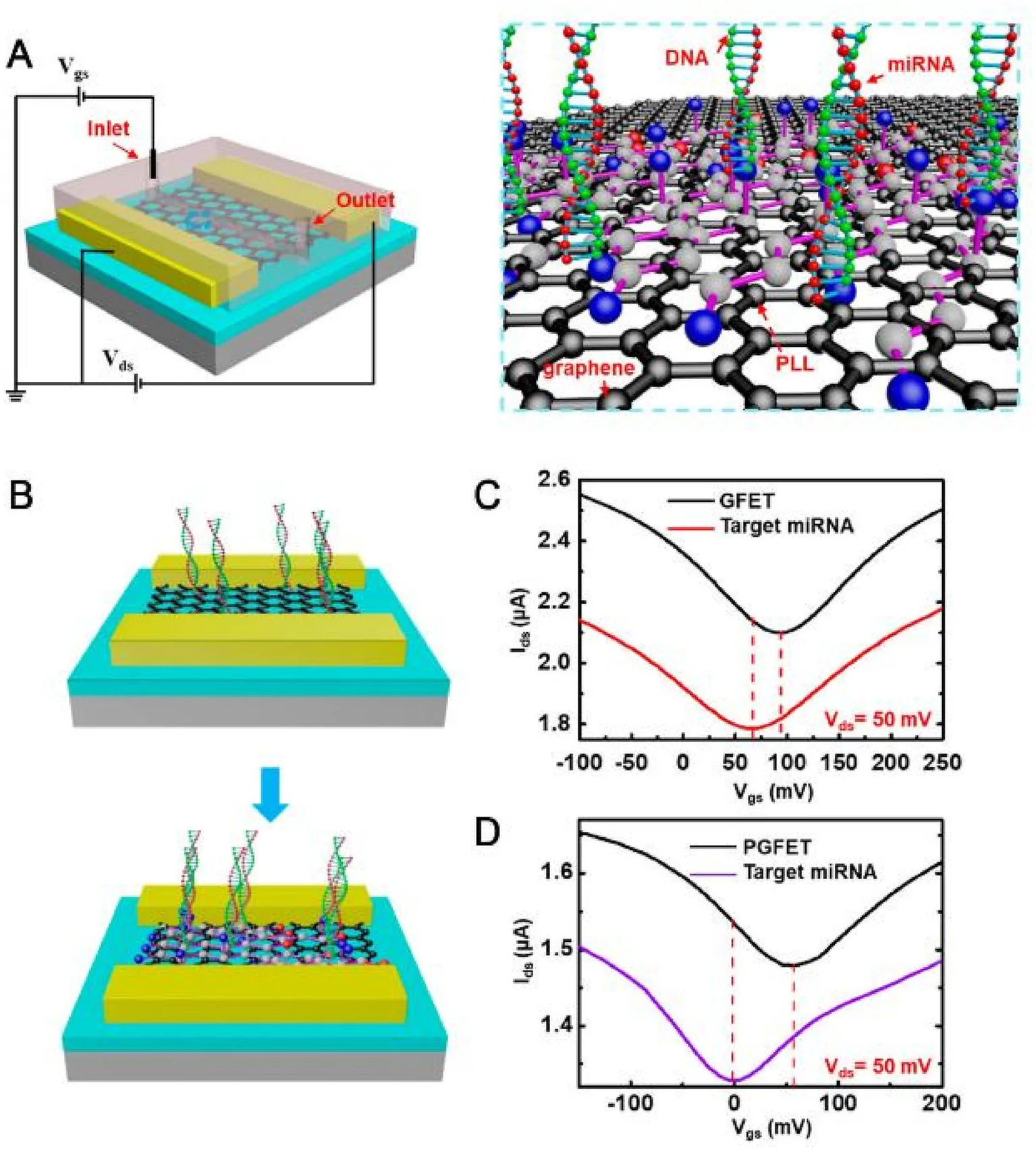
Depicts the field-effect transistor biosensors for Alzheimer’s disease biomarker quantification (97). **(A)** 3D schematic of a graphene-based field-effect transistor biosensor; **(B)** schematic of target miRNA hybridizing with surface-bound DNA probes; **(C)** line graph comparing current versus gate voltage for bare GFET and target miRNA; **(D)** line graph for PGFET and target miRNA highlighting current changes after hybridization.

Current translation efforts focus on lateral flow immunoassays (LFIAs), which provide rapid visual detection of S100B within approximately 15 min without specialized instrumentation. Several LFIA platforms are available in Europe, where S100B has been incorporated into clinical practice guidelines, although they have not received approval from the United States Food and Drug Administration (FDA). While lateral flow assays generally have higher detection limits than laboratory-based electrochemical biosensors, their portability, ease of use, and suitability for point-of-care testing make them the most mature technology for rapid S100B assessment (95, 96). Figure 8 depicts the field-effect transistor biosensors for Alzheimer’s disease biomarker quantification (97).

### 4.1 GFAP biosensors

GFAP biosensor development has accelerated following the United States Food and Drug Administration (FDA) clearance of the Banyan BTI assay, which established a clinical decision threshold of 22 pg./mL. Most GFAP biosensors use nanomaterial-enhanced electrodes incorporating gold nanoparticles, gold nanorods, reduced graphene oxide, carbon nanotubes, or MXenes to improve antibody immobilization, electron transfer, and signal amplification. Electrochemical and optical sensing platforms dominate current research, with MXene-based sensors receiving particular attention because of their high electrical conductivity, large surface area, and favorable surface chemistry for biomolecule attachment (98, 99).

State-of-the-art GFAP biosensors achieve detection limits between 0.01 and 1 pg./mL, well below the FDA clinical threshold, and provide linear detection ranges that cover both normal physiological concentrations and elevated levels observed in severe traumatic brain injury. Assay times typically range from 15 to 90 min, depending on the sensing strategy. Studies have shown that sensor calibration should account for differences between serum and plasma because GFAP concentrations vary between these sample types, making matrix-specific calibration important for accurate clinical measurements (100).

**Figure 9:**
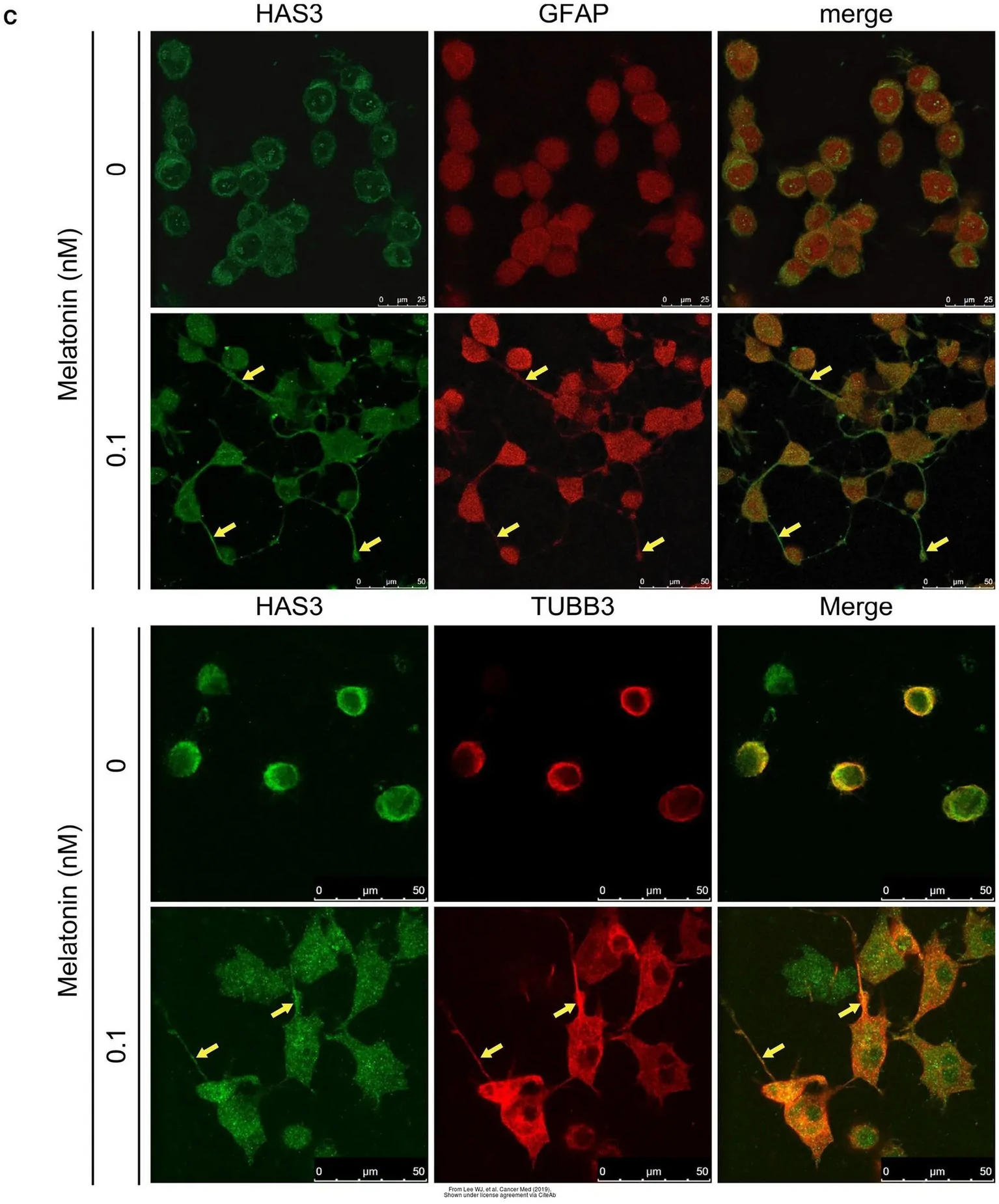
Immunofluorescence staining showing elevated HAS3 expression alongside GFAP astrocyte and TUBB3 neuronal markers in cells treated with 0.1 nM melatonin relative to untreated (0 nM) controls, with yellow arrows highlighting enhanced morphological and biomarker signals upon melatonin exposure (102).

GFAP biosensors have undergone more extensive clinical validation than many other traumatic brain injury biosensors because FDA-approved immunoassays are available as reference methods. Several studies have reported good agreement between biosensor measurements and established laboratory assays across different injury severities. The existing regulatory framework and clinical evidence supporting GFAP provide a strong foundation for translating these biosensors into point-of-care diagnostic devices and future regulatory approval (101). Figure 9 depicts the Immunofluorescence staining showing elevated HAS3 expression alongside GFAP astrocyte and TUBB3 neuronal markers in cells treated with 0.1 nM melatonin relative to untreated (0 nM) controls, with yellow arrows highlighting enhanced morphological and biomarker signals upon melatonin exposure (102).

### 4.2 UCH-L1 biosensors

UCH-L1 biosensors are designed to detect a neuronal injury biomarker that is particularly valuable for evaluating mild traumatic brain injury (TBI). Most platforms use electrochemical sandwich immunoassays with anti-UCH-L1 antibodies immobilized on gold or carbon-based electrodes, often enhanced with gold nanoparticles and carbon nanotubes to improve signal sensitivity. Aptamer-based biosensors have also been developed, offering high specificity, reversible target binding, and improved stability for point-of-care applications (103).

Recent UCH-L1 biosensors have reported detection limits ranging from 0.01 to 10 pg./mL, with linear detection ranges extending from sub-picogram to nanogram concentrations. These performance levels are sufficient to detect the FDA clinical threshold of 327 pg./mL. Sensor selectivity has been improved by using monoclonal antibodies or highly specific aptamers to minimize cross-reactivity with related proteins such as UCH-L3. Although analytical performance is promising, most studies have been limited to buffer solutions or biomarker-spiked serum, with relatively few investigations using clinical samples from TBI patients (104).

**Figure 10:**
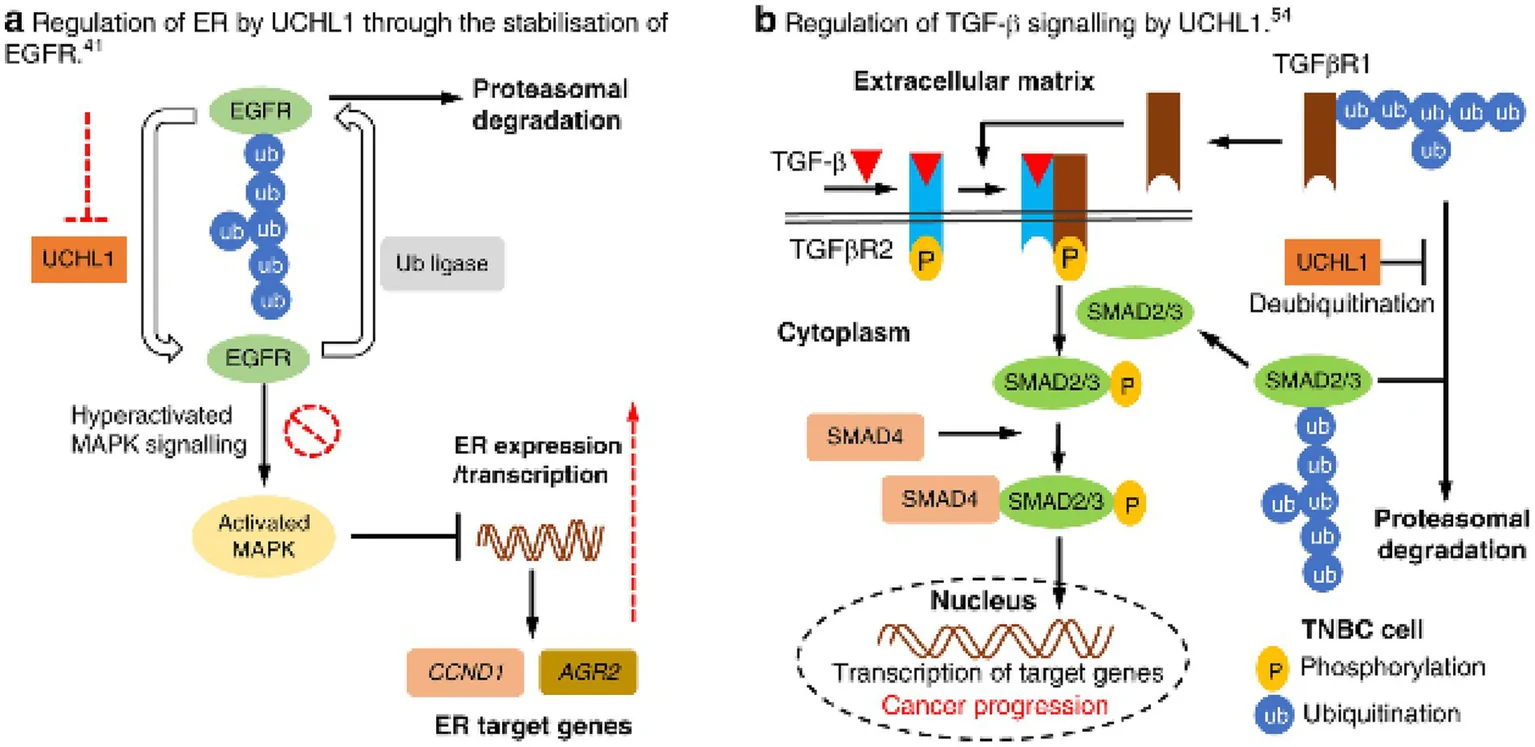
UCHL1 disrupts balanced ubiquitination to stabilize EGFR and suppress ERα/MAPK signaling in breast cancer while also deubiquitinating TGF*β*R1 and SMAD2/3 to sustain oncogenic TGF-β pathway activity in metastatic triple-negative breast cancer, with UCHL1 inhibition driving degradation of these targets to yield anti-tumour effects (106). **(a)** UCHL1 preventing EGFR ubiquitination and degradation, resulting in increased MAPK signaling and suppression of ER gene expression; **(b)** UCHL1 deubiquitinating TGFβR1, promoting SMAD phosphorylation, nuclear translocation, and transcription of genes driving cancer progression.

UCH-L1 benefits from an established regulatory pathway because it is included with GFAP in the FDA-cleared Banyan BTI diagnostic panel. This regulatory precedent supports the development of multiplexed GFAP/UCH-L1 biosensors that simultaneously measure glial and neuronal injury markers. Such integrated platforms are emerging as promising point-of-care diagnostic systems for rapid TBI assessment and clinical decision-making (105). Figure 10 demonstrates the UCHL1 disrupts balanced ubiquitination to stabilize EGFR and suppress ERα/MAPK signaling in breast cancer while also deubiquitinating TGF*β*R1 and SMAD2/3 to sustain oncogenic TGF-β pathway activity in metastatic triple-negative breast cancer, with UCHL1 inhibition driving degradation of these targets to yield anti-tumour effects (106). The biosensor platforms reviewed in this section target serum or plasma GFAP, consistent with the blood-based clinical decision thresholds (22 pg./mL) used in TBI triage, unless CSF is explicitly identified as the analytical matrix.

### 4.3 NfL biosensors

NfL biosensors are designed to detect neurofilament light chain, an axonal injury biomarker that is most useful for monitoring subacute, chronic, and repetitive traumatic brain injury (TBI). Unlike S100B, GFAP, and UCH-L1, NfL is primarily used for long-term assessment rather than acute diagnosis. Because healthy individuals have low circulating NfL concentrations that overlap with those observed in mild TBI, biosensors require high analytical sensitivity and precise quantification in the low picogram-per-milliliter range. Current platforms include electrochemical sensors, graphene field-effect transistors, fluorescence-based systems, nanopore technologies, and digital single-molecule detection approaches developed to approach the performance of the Simoa platform, which remains the analytical reference standard (107–109).

Recent NfL biosensors have achieved detection limits between 0.1 and 10 pg./mL, with several nanomaterial-enhanced electrochemical and fluorescence platforms detecting concentrations below 1 pg./mL. Although these results demonstrate excellent analytical performance, most studies have been limited to buffer solutions or biomarker-spiked serum. Clinical validation remains limited, and relatively few investigations have evaluated NfL biosensors using samples from patients with TBI, reducing the current evidence for routine clinical implementation (110, 111).

**Figure 11:**
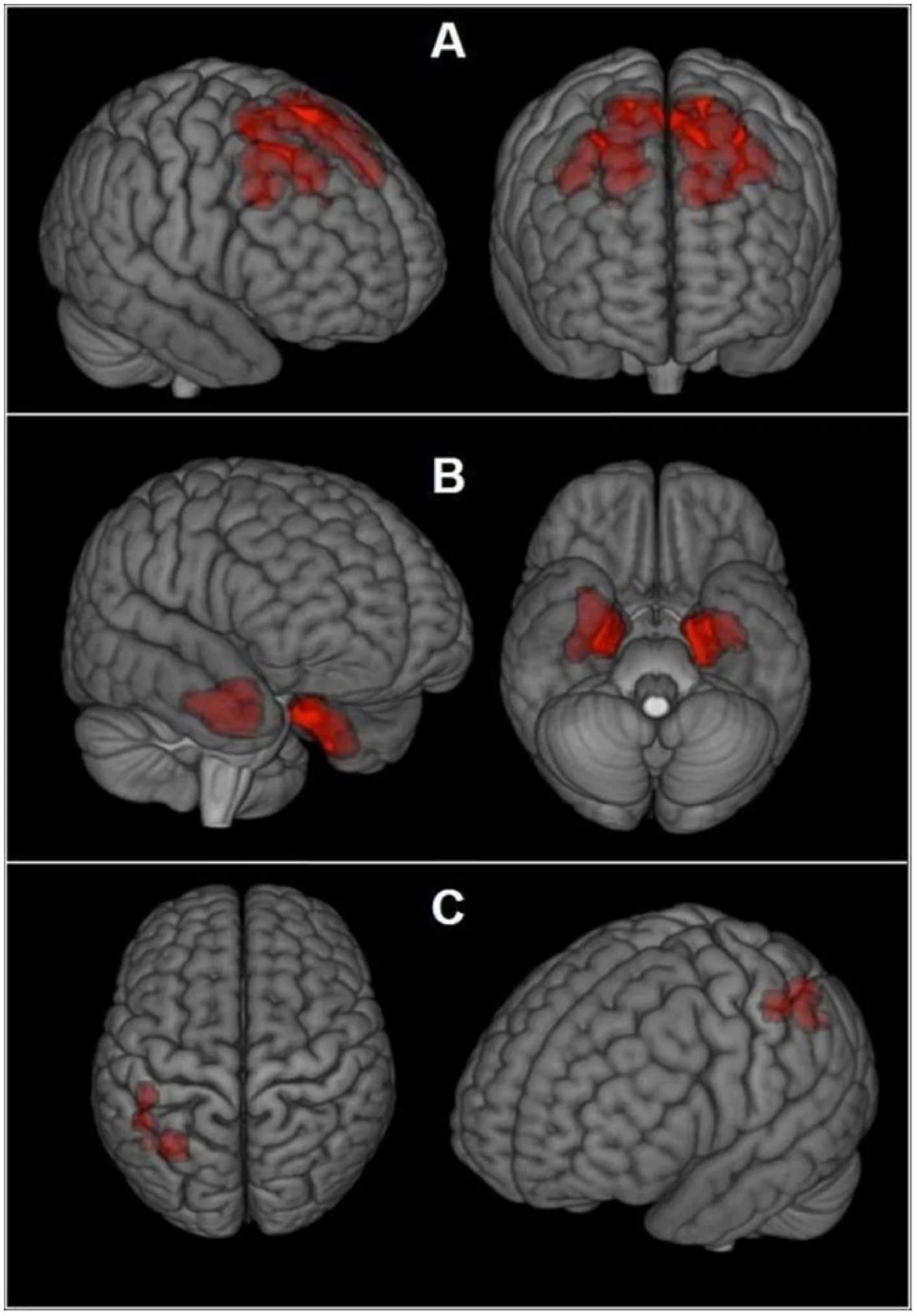
Three-dimensional brain renderings adapted from April 2019 New England Journal of Medicine imaging of former NFL athletes, with red patches marking elevated abnormal disease-associated protein deposits linked to repetitive head trauma, contrasted against healthy control men (image credit: The New England Journal of Medicine via AP) (113). **(A)** frontal lobe activation; **(B)** bilateral occipital and temporal activation; **(C)** localized activation within the left hemisphere — shown as three-dimensional brain renderings adapted from April 2019 New England Journal of Medicine imaging of former NFL athletes.

The translation of NfL biosensors into clinical practice remains at an early stage because no FDA-approved diagnostic test currently exists for NfL. Nevertheless, large clinical studies have established NfL as a valuable biomarker for monitoring axonal injury and recovery following TBI. Portable NfL biosensors have considerable potential for serial monitoring in athletes, military personnel, and other individuals at risk of repeated head injury, providing a practical approach for long-term patient follow-up outside specialized laboratories (112). Figure 11 depicts the Three-dimensional brain renderings adapted from April 2019 *New England Journal of Medicine* imaging of former NFL athletes, with red patches marking elevated abnormal disease-associated protein deposits linked to repetitive head trauma, contrasted against healthy control men (image credit: The New England Journal of Medicine via AP) (113).

### 4.4 Multiplexed and multi-biomarker biosensing platforms

Multiplexed biosensors enable the simultaneous detection of multiple traumatic brain injury (TBI) biomarkers from a single sample, providing more comprehensive diagnostic and prognostic information than single-biomarker assays. Combining biomarkers such as S100B, GFAP, UCH-L1, and neurofilament light chain (NfL) improves assessment because each reflects different aspects of brain injury and exhibits distinct release kinetics. Current multiplexed platforms commonly use multi-electrode arrays, closed bipolar electrodes, or microfluidic cartridges that integrate sample handling and electrochemical detection into compact devices suitable for point-of-care testing (114, 115).

Optical multiplexing approaches include antibody microarrays, quantum dot-based immunoassays, and surface-enhanced Raman spectroscopy (SERS), which distinguish multiple biomarkers using spatial or spectral encoding. These technologies enable simultaneous analysis of several proteins from a single specimen, although some platforms still require laboratory instrumentation. Recent advances in portable fluorescence and electrochemical systems have improved the feasibility of multiplexed testing outside centralized laboratories (115).

Among current developments, simultaneous detection of GFAP and UCH-L1 has attracted the greatest clinical interest because these biomarkers form the basis of the FDA-cleared Banyan BTI diagnostic panel. Recent multiplexed electrochemical biosensors have demonstrated the ability to measure both biomarkers in patient samples with clinically relevant sensitivity, representing an important step toward rapid, portable, and comprehensive TBI diagnosis (114).

### 4.5 AI and machine learning-integrated biosensing platforms

Artificial intelligence (AI) and machine learning (ML) are expanding the capabilities of traumatic brain injury (TBI) biosensors by improving signal analysis, biomarker interpretation, and point-of-care diagnostics. Deep learning models, including convolutional neural networks (CNNs) and recurrent neural networks (RNNs), can analyze complex electrochemical signals such as impedance spectra, voltammograms, and chronoamperometric responses with greater accuracy than conventional signal-processing methods. These models reduce the effects of background noise and matrix interference, enabling more precise estimation of biomarker concentrations (116, 117).

Machine learning algorithms, including random forests, gradient boosting, and support vector machines, have also been applied to multiplex biomarker panels to classify injury severity, predict computed tomography (CT) findings, and estimate clinical outcomes. By integrating information from multiple biomarkers, AI-assisted models generally achieve higher diagnostic accuracy than single-biomarker approaches. This strategy supports more reliable clinical decision-making and complements the development of multiplexed biosensor platforms (114, 118).

**Figure 12:**
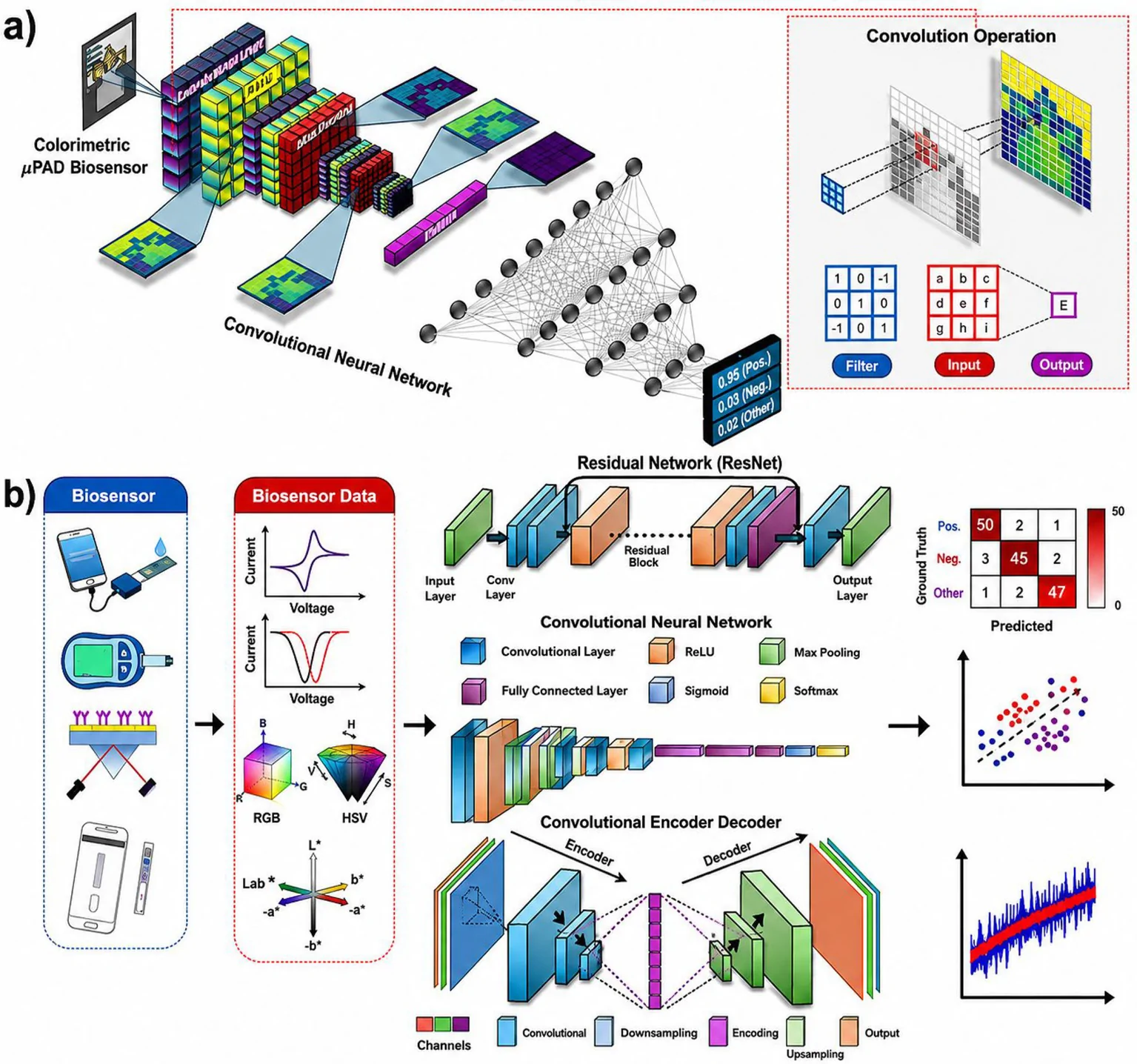
The impact of artificial intelligence on the development of biosensor technology (116). **(a)** colorimetric μPAD biosensor inputting data into a convolutional neural network with an inset visualizing a convolution operation on image data; **(b)** different biosensor devices and types of biosensor data (RGB, HSV, and various graphs) processed through residual networks, convolutional neural networks, and convolutional encoder-decoders, resulting in output graphs and a confusion matrix.

AI further enhances point-of-care testing by enabling quantitative analysis of lateral flow and colorimetric biosensors using smartphone images. Convolutional neural networks can measure faint test-line intensities with accuracy approaching dedicated laboratory readers, allowing portable biosensors to generate quantitative results without specialized instrumentation. These advances support the development of accessible, intelligent biosensing systems for rapid TBI diagnosis and patient monitoring (114, 115). Figure 12 demonstrates the Impact of Artificial Intelligence on the Development of Biosensor Technology (116).

Table 4 summarizes the molecular properties, biological origin, temporal kinetics, regulatory thresholds, and primary clinical applications of the four major TBI biomarkers: S100B, GFAP, UCH-L1, and NfL. It establishes the biological foundation upon which all biosensor performance evaluations in the review are anchored. Without this reference, the clinical relevance of reported detection limits and assay windows cannot be properly interpreted.

**Table 4:** Comparative analytical performance of nanomaterial-based biosensors for TBI biomarkers (197–202, 221–224).

Biomarker	Platform type	Nanomaterial	Recognition element	LOD	Linear range	Assay time	Sample matrix	Clinical threshold	Threshold surpassed?
S100B	Lateral flow immunoassay (LFIA)	Colloidal gold NPs	Anti-S100B antibody	4.6 pg./mL	0.01–2,000 pg./mL	~15 min	Spiked serum	100–150 pg./mL (SNC)	Yes
S100B	Time-resolved fluorescence immunochromatography	Anti-S100B monoclonal Ab	Anti-S100B Ab	12.6 pg./mL	15.6–1,000 pg./mL	~13 min	Spiked serum	100–150 pg./mL	Yes
S100B	Electrochemical (EIS)	Gold NPs + CNTs	Anti-S100B Ab	~10–50 fg/mL	fg/mL–ng/mL	20–60 min	Buffer / spiked serum	100–150 pg./mL	Yes
S100B	Optical (LSPR)	Gold nanorods	Anti-S100B Ab	~0.5–5 pg./mL	pg/mL–ng/mL	30–60 min	Buffer	100–150 pg./mL	Yes
GFAP	GFET (graphene field-effect transistor)	Monolayer graphene	Anti-GFAP Ab	20 fg/mL (400 aM)	fg/mL–ng/mL	<30 min	Patient plasma	22 pg./mL (FDA-BTI)	Yes
GFAP	Electrochemical (EIS, PEI-graphene SPE)	rGO / PEI-graphene	Anti-GFAP Ab	~1 pg./mL	1 pg./mL–100 ng/mL	30–60 min	Buffer/spiked serum	22 pg./mL	Yes
GFAP	SERS (Ag nanorod thin film)	Ag nanorod nSTF	Anti-GFAP Ab	sub-pg/mL	pg/mL–ng/mL	~45 min	Blast-exposed rat serum	22 pg./mL	Yes
GFAP	Electrochemical (rGO/MoS₂/AgNPs)	rGO + MoS₂ + AgNPs	Anti-GFAP Ab	<1 pg./mL	pg/mL–ng/mL	30–60 min	CSF	22 pg./mL	Yes
UCH-L1	Electrochemical sandwich immunosensor	Gold NPs + CNTs	Anti-UCH-L1 Ab	0.01–10 pg./mL	sub-pg/mL–ng/mL	30–60 min	Buffer / spiked serum	327 pg./mL (FDA-BTI)	Yes
UCH-L1	Aptasensor (electrochemical)	Gold electrode + AuNPs	UCH-L1 aptamer	~1–5 pg./mL	pg/mL range	20–40 min	Buffer	327 pg./mL	Yes
UCH-L1	SPR-based	Gold film	Anti-UCH-L1 Ab	~1–10 pg./mL	pg/mL–ng/mL	30–60 min	Buffer	327 pg./mL	Yes
NfL	Electrochemical immunosensor (ZrO₂@La₂O₃)	MOF-derived ZrO₂@La₂O₃	Anti-NfL Ab	5.21 pg./mL	pg/mL range	>14 h (preparation)	Buffer	No FDA threshold; SIMOA reference LOD ~ 1.6 pg./mL	Comparable
NfL	GFET	Graphene	Anti-NfL Ab	sub-pg/mL	pg/mL range	<30 min	Buffer/spiked	No FDA threshold	Reference standard required
NfL	Electrochemical (sandwich, SPCE + MBs)	Magnetic microbeads + SPE	Anti-NfL Ab + HRP-Ab	~0.1–1 pg./mL	0.1–100 pg./mL	30–60 min	Cell culture / buffer	No FDA threshold	Promising
NfL	SIMOA (reference standard)	—	Ab-coated nanobead	~1.6 pg./mL	pg/mL range	2–4 h (lab)	Serum / plasma	Clinical reference	Benchmark

SNC, Scandinavian Neurotrauma Committee; FDA-BTI, FDA-cleared Banyan Brain Trauma Indicator; rGO, reduced graphene oxide; nSTF, nanostructured thin film; MBs, magnetic microbeads; SPCE, screen-printed carbon electrode; EIS, electrochemical impedance spectroscopy; SPR, surface plasmon resonance; GFET, graphene field-effect transistor; SERS, surface-enhanced Raman spectroscopy; Ab, antibody; AuNPs, gold nanoparticles; CNTs, carbon nanotubes; LSPR, localized surface plasmon resonance; LOD, limit of detection.

## 5 Integrative comparative analysis

### 5.1 Quantitative cross-platform benchmarking

The clinical value of a traumatic brain injury (TBI) biosensor depends on whether its analytical performance matches the concentration range of the target biomarker. For S100B and GFAP, biosensors require low detection limits to distinguish healthy individuals from patients with mild TBI, while UCH-L1 requires accurate measurement around its higher clinical threshold. NfL presents the greatest challenge because its concentrations in healthy individuals overlap with those observed during the early stages of mild TBI, demanding both high sensitivity and excellent measurement precision. Therefore, biosensor performance should be evaluated using clinically relevant detection limits, linear dynamic range, and quantitative accuracy rather than analytical sensitivity alone (119–121).

Across all four biomarkers, nanomaterial-enhanced electrochemical biosensors consistently provide the lowest detection limits, often well below clinically required thresholds. Surface plasmon resonance (SPR) and localized surface plasmon resonance (LSPR) platforms also demonstrate high sensitivity while offering label-free, real-time measurements. In contrast, lateral flow and colorimetric biosensors generally exhibit higher detection limits that approach clinical decision thresholds, reducing their analytical margin despite their simplicity and portability. Although laboratory-based biosensors achieve excellent analytical performance, many have been validated only in buffer solutions or biomarker-spiked serum, highlighting the need for broader validation using clinical samples before routine implementation in TBI diagnosis (122–124).

### 5.2 Structure-performance relationships, validation, and manufacturability

The performance of traumatic brain injury (TBI) biosensors is strongly influenced by the choice of nanomaterials and recognition elements. Gold nanoparticles, carbon nanotubes, graphene derivatives, and MXenes consistently improve analytical sensitivity by increasing surface area, enhancing electron transfer, and providing efficient platforms for biomolecule immobilization. Among these materials, Ti₃C₂T_x_ MXene has shown particularly strong performance because of its high conductivity, large active surface area, and favorable surface chemistry. Aptamers are emerging as an attractive alternative to antibodies because they offer greater chemical stability, controlled surface modification, sensor regeneration, and lower production costs while maintaining comparable analytical performance for many TBI biomarkers (125, 126).

Despite excellent analytical results, clinical validation remains limited. Most published biosensors have been evaluated using buffer solutions or biomarker-spiked serum, while only a small proportion have been tested with samples from patients with traumatic brain injury and compared against established clinical reference methods. This gap limits confidence in the clinical applicability of many reported platforms. Future studies should place greater emphasis on patient-based validation, standardized statistical comparisons with approved immunoassays, and assessment of biosensor performance under real clinical conditions (127, 128).

Manufacturing cost and scalability are critical factors for clinical translation. Screen-printed electrochemical electrodes offer the greatest potential for large-scale production because they are compatible with established manufacturing processes used for commercial glucose test strips. However, reproducible deposition of nanomaterials and consistent sensor fabrication remain important engineering challenges. Recognition elements also influence production cost, with chemically synthesized aptamers offering a substantially lower-cost alternative to monoclonal antibodies. Addressing these manufacturing and validation challenges will be essential for the widespread adoption of portable TBI biosensors in clinical practice (129, 130).

## 6 Regulatory, economic, and implementation considerations

The clinical translation of traumatic brain injury (TBI) biosensors depends on regulatory approval, standardized measurement, and clinical validation. In the United States, the FDA clearance of the Banyan Brain Trauma Indicator and the Abbott i-STAT TBI panel established regulatory pathways for blood-based point-of-care biomarker testing, particularly for GFAP and UCH-L1. Although many biosensors demonstrate strong analytical performance, most lack the large prospective clinical studies required to establish diagnostic accuracy in patients with acute TBI. Another challenge is the absence of standardized reference materials and calibration methods, leading to differences in biomarker measurements across analytical platforms and limiting the direct application of common diagnostic thresholds (131, 132).

Point-of-care TBI biosensors also offer substantial economic benefits by reducing unnecessary computed tomography (CT) scans and improving patient triage. Biomarker-guided assessment has the potential to decrease imaging costs, shorten emergency department evaluation, and support earlier identification of patients who require specialized neurosurgical care. In low- and middle-income countries, where access to CT imaging is limited, portable biosensors could improve referral decisions and optimize the use of healthcare resources. These advantages depend on the availability of affordable, accurate, and clinically validated biosensor platforms (133).

Wearable and continuous monitoring systems represent an emerging direction for TBI management by enabling repeated measurement of biomarkers in sweat, tears, or interstitial fluid. However, several technical challenges remain, including biofouling of sensor surfaces, calibration drift during prolonged use, and uncertain correlations between biomarker concentrations in alternative biofluids and blood. Addressing these issues through improved sensor materials, calibration strategies, and clinical validation will be essential before wearable TBI biosensors can become reliable tools for long-term patient monitoring (134, 135).

## 7 Regulatory, economic, and implementation considerations

The clinical translation of traumatic brain injury (TBI) biosensors depends on regulatory approval, standardized measurement, and clinical validation. In the United States, the FDA clearance of the Banyan Brain Trauma Indicator and the Abbott i-STAT TBI panel established regulatory pathways for blood-based point-of-care biomarker testing, particularly for GFAP and UCH-L1. Although many biosensors demonstrate strong analytical performance, most lack the large prospective clinical studies required to establish diagnostic accuracy in patients with acute TBI. Another challenge is the absence of standardized reference materials and calibration methods, leading to differences in biomarker measurements across analytical platforms and limiting the direct application of common diagnostic thresholds (136, 137).

Point-of-care TBI biosensors also offer substantial economic benefits by reducing unnecessary computed tomography (CT) scans and improving patient triage. Biomarker-guided assessment has the potential to decrease imaging costs, shorten emergency department evaluation, and support earlier identification of patients who require specialized neurosurgical care. In low- and middle-income countries, where access to CT imaging is limited, portable biosensors could improve referral decisions and optimize the use of healthcare resources. These advantages depend on the availability of affordable, accurate, and clinically validated biosensor platforms.

Wearable and continuous monitoring systems represent an emerging direction for TBI management by enabling repeated measurement of biomarkers in sweat, tears, or interstitial fluid. However, several technical challenges remain, including biofouling of sensor surfaces, calibration drift during prolonged use, and uncertain correlations between biomarker concentrations in alternative biofluids and blood. Addressing these issues through improved sensor materials, calibration strategies, and clinical validation will be essential before wearable TBI biosensors can become reliable tools for long-term patient monitoring (138). Cross-disciplinary nanomaterial research delivers tunable interfacial electron transport and functional substrate coatings to boost biosensor transduction, covering p–p orbital hybridization engineering, MXene/LIG flexible composite films, thiophene ionic liquid capture layers, shear-dependent nanoparticle protein corona behaviour, and Zn–I₂ interfacial nanoarchitectonic design (139, 140, 142–144). Diverse artificial intelligence algorithms streamline multiplexed biomarker signal processing, medical imaging segmentation, diagnostic uncertainty calibration, hemodynamic prediction and portable hardware topology optimisation for point-of-care platforms (141, 145–148). Neural network modelling frameworks decode complex brain activity patterns by integrating resting and task-evoked neural dynamics to quantify pathological brain signalling states linked to nervous system injury (149). High-definition transcranial direct current stimulation investigations characterise altered cerebral hemodynamics, modified resting-state network connectivity and cortical activation shifts under motor fatigue and sensorimotor dysfunction (150, 151, 153, 190, 191). Trace-element mediated neurodegeneration research establishes fundamental chemical links between metallic micronutrient dysregulation and progressive neuronal tissue damage relevant to brain injury biomarker profiling (154). Atomically precise low-dimensional semiconductor fabrication produces high-quality black phosphorus nanoribbons with uniform edge geometry ideal for ultrasensitive optical and electrochemical biosensing interfaces (152). Graph-theoretic and meta-analytic clinical studies further validate how disrupted whole-brain network topology correlates with impaired sensorimotor function following neural tissue insult, supporting multimodal TBI diagnostic stratification (153, 155, 156).

Data-driven multi-vehicle perception and ML trajectory forecasting models yield robust out-of-distribution motion sequence predictions under variable behavioral input distributions (157, 158); this sequential forecasting paradigm is directly transferable to temporal TBI neurophysiological signal trend prediction. Edge intelligence pre-deployment and location-aware ML resource allocation algorithms optimize distributed edge node compute scheduling for sparse, geographically dispersed data sampling pipelines (159), a critical framework for decentralized pre-hospital TBI patient monitoring edge networks. Constrained multi-objective ML scheduling systems resolve conflicting computational and operational resource constraints within high-complexity sequential workflow pipelines (160, 161), analogous to multi-stakeholder clinical resource allocation for acute TBI triage care pathways. End-to-end automated machine learning (AutoML) emergency inference pipelines enable high-fidelity target state tracking under non-stationary dynamic boundary conditions (162), with analogous utility for monitoring unstable intracranial pressure dynamics in severe TBI patients.

Reconfigurable intelligent surface (RIS)-augmented integrated sensing and communication ML frameworks implement constrained multi-objective signal optimization for low-signal, extended-range perceptual target tasks (163), applicable to noisy wearable biosignal data collection for ambulatory mild TBI monitoring. Sample-efficient temporal difference RL with human-in-the-loop feedback policy alignment mechanisms improve large language model (LLM) factual grounding and sequential decision generalization under sparse reward constraints (164, 165); this human-feedback alignment protocol can be repurposed to calibrate ML TBI prognostic models against clinician gold-standard diagnostic judgements. ML compiler automated hyperparameter tuning frameworks minimize runtime computational overhead during high-volume model inference (166), optimizing latency for real-time edge-executed TBI diagnostic classification models.

Constrained cross Q-learning multi-agent resource allocation schemes harmonize heterogeneous throughput constraints for user-centric distributed optical wireless sensing networks (167), transferable to balancing bandwidth and compute loads for multi-modal TBI ICU monitoring sensor arrays. Spatial channel attention neural modules boost fine-grained 3D contextual feature extraction within neural radiance field (NeRF) volumetric reconstruction pipelines (168), offering state-of-the-art feature enhancement for 3D volumetric TBI brain lesion segmentation tasks. Edge neural network inference pruning and quantization optimization techniques reduce on-device latency and power consumption for embedded lightweight ML deployments (169), key for battery-constrained pre-hospital wearable TBI monitoring hardware systems.

Data-driven algebraic regulator ML control architectures achieve stable trajectory state tracking under severely limited labelled training sample regimes (170), a high-priority capability for low-data rare-cohort TBI patient subgroup predictive modelling. Collaborative multi-modal perceptual ML fused with task offloading optimization streamline high-dimensional biosignal data exchange across 6G air-ground integrated edge communication infrastructures (171), supporting cross-site decentralized TBI big data cohort aggregation. Two-stage hierarchical deep reinforcement learning (DRL) architectures optimize latency-bounded cooperative edge content caching policies for time-critical vehicular edge networks (172), generalizable to low-latency urgent neuroimaging data caching for emergency TBI trauma care workflows.

Semantic grounded category-level robotic grasping neural networks extract hierarchical structured high-dimensional visual embeddings for precision physical state manipulation (173), with transferable feature hierarchy design principles for hierarchical TBI neuroimage biomarker segmentation. Compression-aware adaptive stream query ML frameworks maximize inference throughput across heterogeneous parallel compute hardware (174), accelerating batch processing of high-volume continuous TBI intracranial sensor time-series data. Stability-regularized DRL scheduler architectures mitigate noisy redundant data transmission artefacts over time-variant stochastic Gilbert-Elliot wireless channel noise profiles (175), improving signal fidelity for remote long-term post-discharge TBI neurophysiological state monitoring.

Fine-grained boundary-aware self-supervised perception neural networks enhance low-contrast edge and lesion boundary feature extraction in unlabelled indoor depth estimation pipelines (176), a core ML feature learning strategy for ambiguous mild TBI diffuse axonal injury imaging segmentation. Graph attention network (GAT)-embedded mobility-aware ML scheduling models optimize dual-link heterogeneous resource orchestration across unified terrestrial and non-terrestrial communication ecosystems (177), enabling cross-regional multi-site TBI cohort data harmonization. Optical flow guided recursive predictive neural architectures refine sub-pixel precision fluid dynamic field quantification via particle image velocimetry benchmarking (178), transferable to ML modelling of cerebral blood flow haemodynamic dynamics post-TBI injury.

Hierarchical multi-layer adversarial security ML frameworks mitigate untrusted third-party task offloading risks in resource-scarce edge computing environments (179), critical for protecting sensitive HIPAA-regulated TBI patient clinical and neuroimaging datasets. Distance-constrained fair reinforcement learning resource allocation mechanisms balance utilitarian and egalitarian resource distribution objectives for geographically dispersed crowdsensing task cohorts (180), establishing fairness guardrails for multi-site TBI clinical trial ML data sampling protocols. Multi-agent DRL pipelines embedded with ethical disaster relief fairness constraints automate equitable edge computing task partitioning for high-stakes emergency distributed systems (181), definable for equitable acute TBI trauma care resource triage ML decision pipelines.

Heterogeneous agent-based vehicle-road-cloud ML orchestration architectures standardize cross-domain policy and resource normalization for connected intelligent distributed system ecosystems (182), supporting unified cross-modal TBI clinical, wearable and imaging data lake governance. One-shot federated clustering ML algorithms perform unsupervised cohort pattern mining on non-IID (non-independent and identically distributed) siloed datasets without centralized raw patient data pooling (183), solving core data privacy constraints for multi-center TBI retrospective cohort ML analysis. Mixture-of-Experts (MoE) collaborative distributed inference frameworks drastically reduce edge-side computational burden for large foundation model execution on constrained peripheral hardware (184), enabling foundation model deployment for complex multi-modal TBI prognostic risk scoring at clinical edge terminals.

Differential privacy-preserving model identity alignment protocols eliminate longitudinal user feature tracking vulnerabilities in continuous sequential ML inference workflows (185), securing longitudinal repeated TBI patient prognostic monitoring pipelines against sensitive phenotype leakage. Multi-user concurrent oblivious RAM ML security architectures enforce end-to-end access control for shared cloud-hosted clinical datastores (186), hardening centralized TBI research biobanks against unauthorized data exfiltration. Constraint-informed motion planning neural models resolve multi-agent collision and movement conflicts for clustered automated guided vehicle fleets in confined operational spaces (187), with analogous constraint logic for multi-criteria safe clinical workflow scheduling in TBI neurocritical care units. Multi-agent DRL closed-loop control architectures jointly optimize the tri-objective tradeoff of operational safety, task execution efficiency, and subjective user comfort during cooperative autonomous sequential manoeuvring (188); this multi-criteria balancing ML paradigm is directly adaptable to optimizing TBI patient sedation, intracranial pressure management, and long-term functional recovery multi-target treatment control (225–227).

## 8 Conclusion

Traumatic brain injury (TBI) remains a major global diagnostic challenge, especially where rapid neuroimaging is unavailable. Nanomaterial-enabled biosensors incorporating gold nanoparticles, graphene derivatives, carbon nanotubes, and MXenes have significantly improved detection of key biomarkers (S100B, GFAP, UCH-L1, and NfL), achieving clinically relevant or superior detection limits and enabling rapid, portable testing. However, clinical translation is constrained not by analytical sensitivity alone but by limited validation in real patient cohorts, as most platforms remain tested in buffer or spiked matrices. Future progress depends on standardized calibration, reproducible manufacturing, and robust clinical studies across injury severities, alongside multiplexed biosensing and AI-driven interpretation to improve diagnostic accuracy and decision support. Regulatory pathways, scalable fabrication, and cost reduction remain essential for deployment in both high- and low-resource settings. While GFAP and UCH-L1 assay approvals provide regulatory precedent, broader adoption of nanomaterial biosensors will require standardized reference materials, improved inter-laboratory reproducibility, and lower-cost recognition strategies such as aptamers. Coordinated efforts across engineering, clinical neuroscience, and regulatory science are necessary to translate these platforms from high-performance laboratory systems into reliable point-of-care TBI diagnostics.
